# Bitter Taste Receptors in Bacterial Infections and Innate Immunity

**DOI:** 10.1002/iid3.70232

**Published:** 2025-07-25

**Authors:** Erin Rudolph, Hannah Dychtenberg, Austin Pozniak, Priyanka Pundir

**Affiliations:** ^1^ Department of Molecular and Cellular Biology, College of Biological Science University of Guelph Guelph Ontario Canada

**Keywords:** bacterial infection, bitter taste receptors, GPCRs, innate immunity, mucosal immunity, quorum sensing, TAS2Rs, therapeutic targets

## Abstract

**Background:**

Bitter taste receptors (TAS2Rs), originally identified for their role in gustation, are now recognized for their functions in extraoral tissues, particularly in innate immune responses. TAS2Rs detect bacterial quorum sensing molecules (QSMs) and other metabolites, enabling the host to sense and respond to pathogenic threats across mucosal surfaces.

**Objective:**

This review synthesizes current knowledge of TAS2Rs in the context of bacterial infection, emphasizing their mechanisms of immune modulation, genetic polymorphisms, tissue‐specific expression, and therapeutic potential.

**Methods:**

A comprehensive literature review was conducted, incorporating in vitro, ex vivo, and in vivo studies investigating TAS2R expression, signaling pathways, and immune functions in response to bacterial pathogens across respiratory, gastrointestinal, and oral tissues.

**Results:**

TAS2Rs detect bacterial QSMs, triggering calcium signaling cascades, nitric oxide (NO) release, antimicrobial peptide secretion, and cytokine responses. In respiratory epithelium, TAS2R38 and TAS2R14 modulate mucociliary clearance and NO‐mediated bacterial killing. In the oral cavity, TAS2R14 and TAS2R38 influence cytokine production, bacterial uptake, and antimicrobial responses. Intestinal TAS2Rs regulate host defense via genotype‐specific pathways, as seen with TAS2R10 and TAS2R43. Polymorphisms in TAS2Rs affect infection susceptibility and immune responses, with implications for diseases like cystic fibrosis, chronic rhinosinusitis, dental caries, and periodontitis. Notably, TAS2R‐mediated responses are highly tissue‐ and bacteria‐dependent, with distinct signaling and outcomes observed depending on the pathogen and the local immune environment.

**Conclusions:**

TAS2Rs play an essential role in host‐pathogen interactions across multiple mucosal surfaces. Their ability to detect bacterial signals and activate innate immune defenses positions them as promising therapeutic targets. Future studies should focus on in vivo validation, genetic diversity, and receptor‐ligand specificity using emerging tools like cryo‐electron microscopy and transgenic models.

**Abbreviations**
3‐oxo‐C_12_‐HSL: *N*‐3(‐oxo‐dodecanoyl)‐L‐homoserine lactone
AHL: acyl‐homoserine lactone
ALI: air‐liquid interface
C_4_‐HSL: *N*‐butanoyl‐L‐homoserine lactone
cAMP: cyclic adenosine monophosphate
CBF: ciliary beat frequency
CF: cystic fibrosis
cGMP: cyclic guanosine monophosphate
CGRP: calcitonin gene‐related peptide
CRS: chronic rhinosinusitis
Cryo‐EM: cryo‐electron microspcopy
CSPs: competence stimulating peptides
DAG: diacylglycerol
GECs: gingival epithelial cells
GPCRs: G protein‐coupled receptors
hBD‐2: human beta‐defensin‐2
HBE: human bronchial epithelial cells
HHQ: 4‐hydroxy‐2‐heptyquinolone
IL: interleukin
IP_3_
: inositol 1,4,5‐triphosphate
LPS: lipopolysaccharide
NO: nitric oxide
PI3K: phosphoinositide 3‐kinase
PIP_2_
: phosphatidylinositol 4,5‐bisphosphate
PLC: phospholipase C
PQS: 2‐heptyl‐3‐hydroxy‐4‐quinolone, Pseudomonas quinolone signal
QSMs: quorum sensing molecules
SCC: solitary chemosensory cells
SNPs: single‐nucleotide polymorphisms
SP: substance P
TAS2Rs: bitter taste receptors
TNF: tumor necrosis factor
TRPM5: transient receptor potential cation channel subfamily M member 5

## 1 Introduction

Infectious diseases constitute major threats to global health, causing significant illness and loss of life around the world [1, 2]. Despite advances in modern medicine, infectious agents like bacteria, viruses, fungi, and parasites put immense pressure on healthcare systems [3]. Urgent infectious threats underscore the need for targeted and innovative healthcare strategies. By gaining a deeper understanding of how these diseases develop, progress, and interact with host immunity, we can uncover critical insights. These findings can pave the way for more precise treatments and prevention methods, ultimately leading to better health outcomes for individuals and communities around the world.

Among the body's many defense mechanisms, taste receptors have emerged as an unexpected yet significant player. The taste 2 receptor family, TAS2Rs, originally identified for their role in detecting bitter compounds [4], has garnered increasing attention for their functions beyond taste perception, particularly in immune modulation and cellular signaling across tissues. Recent studies have expanded our understanding of TAS2Rs in infection and immune responses. Building on these advancements, this review synthesizes current knowledge of TAS2Rs in infection, examining their diverse functions, signaling pathways, genetic polymorphisms, and structural characteristics. By integrating these aspects, we highlight TAS2Rs as promising therapeutic targets in infectious diseases.

While previous reviews have primarily focused on the role of TAS2Rs in specific sites such as the upper respiratory tract or in the context of a few bacterial species, our review offers a broader perspective by exploring TAS2R function across multiple mucosal surfaces, including the respiratory tract, oral cavity, and intestines. By examining a diverse range of bacterial pathogens and highlighting how TAS2Rs contribute to innate immune defenses in different tissue environments, we provide a more comprehensive view of the emerging role of these receptors in host‐pathogen interactions. This approach not only underscores the versatility of TAS2Rs in immune surveillance but also identifies common and site‐specific mechanisms that may be leveraged for therapeutic development.

### 1.1 Bitter Taste Receptors (TAS2Rs)

Bitter taste perception plays a vital role in survival by detecting and signaling the presence of bitter compounds [5]. TAS2Rs are G protein‐coupled receptors (GPCRs) that range in length from about 290–330 amino acids, possess seven transmembrane helices (TM1–TM7), and contain a short extracellular N‐terminal and intracellular C‐terminal [6]. The amino acids located in the transmembrane domains are highly conserved across TAS2Rs and differ from traditional Class A GPCRs [6]. Furthermore, TAS2Rs have high sequence similarity in intracellular loops, with greater variation in extracellular loop sequences [6].

**Figure 1:**
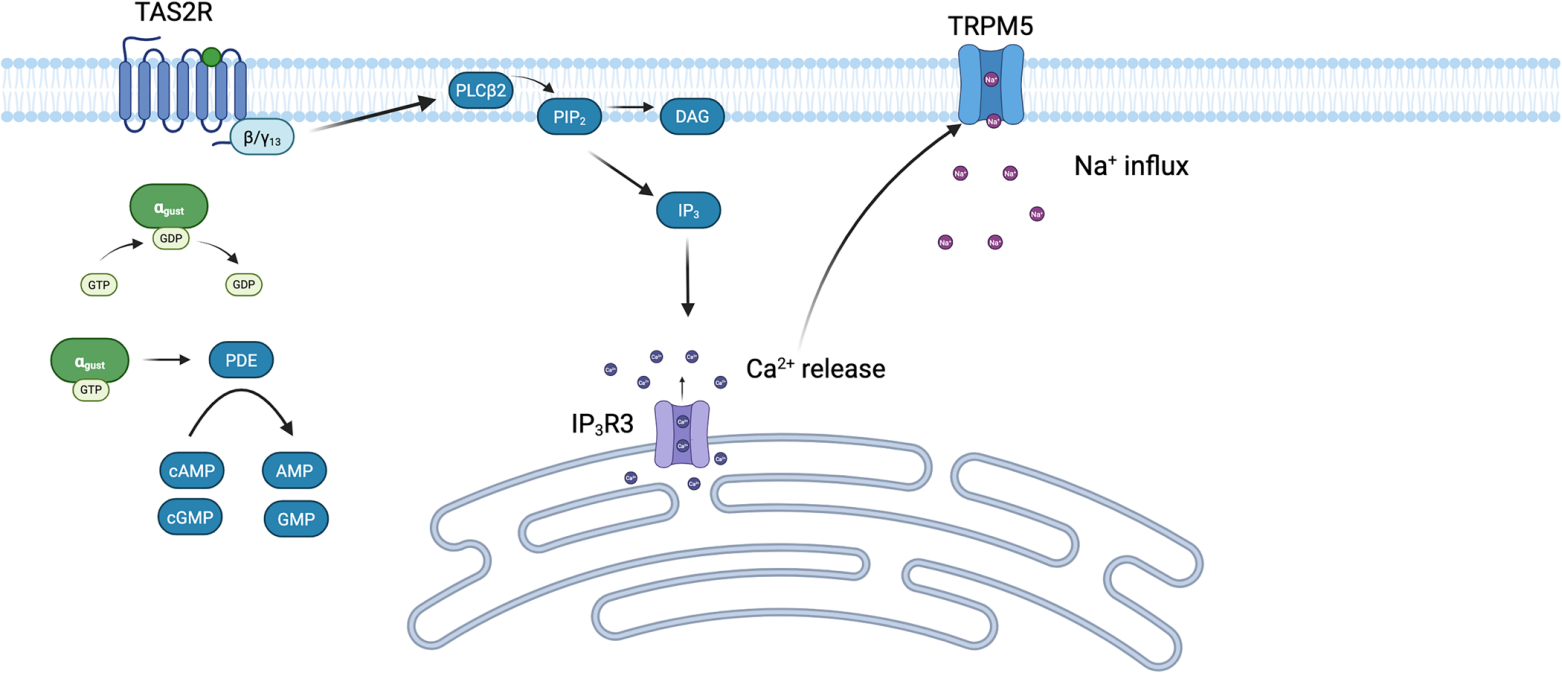
TAS2R activation and underlying signaling mechanism. A bitter compound binds to the TAS2R, resulting in G_αgust_ activation via the exchange of GDP for GTP. G_αgust_ activates an intracellular phosphodiesterase which breaks down cyclic adenosine monophosphate (cAMP) and cyclic guanosine monophosphate (cGMP), decreasing their intracellular levels. The βγ13 subunit of the G protein activates PLCβ2, which cleaves phosphatidylinositol 4,5‐bisphosphate (PIP_2_) into diacylglycerol (DAG) and inositol 1,4,5‐triphosphate (IP_3_). IP_3_ activates IP_3_R3 located on the plasma membrane of the smooth endoplasmic reticulum, triggering Ca^2+^ release. Ca^2+^ binds and activates the TRPM5 (transient receptor potential cation channel subfamily M member 5) ion channel, resulting in Na^+^ influx and membrane depolarization. The figure was created with Biorender.

TAS2R signaling is mediated by a heterotrimeric G protein composed of G_αgust_ and βγ13 subunits (Figure 1) [7, 8]. βγ13 activates the phospholipase C (PLC) isoform, PLCβ2, which cleaves phosphatidylinositol 4,5‐bisphosphate (PIP_2_) into diacylglercol (DAG) and inositol 1,4,5‐triphosphate (IP_3_) [7, 8]. IP_3_ subsequently activates IP_3_R3, triggering the release of calcium (Ca^2+^) from intracellular stores [9]. The released Ca^2+^ ions activate transient receptor potential cation channel subfamily M member 5 (TRPM5), resulting in Na^+^ influx and membrane depolarization [9]. Simultaneously, there is a reduction in the nucleotides cyclic adenosine monophosphate (cAMP) and cyclic guanosine monophosphate (cGMP), believed to be mediated by G_αgust_‐dependent activation of phosphodiesterase [7].

Although TAS2Rs were once thought to be confined to the oral cavity, growing evidence now reveals that these sensory receptors are expressed in numerous extraoral tissues and immune cells (Table 1) [10, 11, 12, 13, 14, 15, 16, 17, 18, 19, 20, 21, 22, 23, 24, 25, 26, 27, 28, 29, 30, 31, 32, 33, 34, 35, 36, 37, 38, 39, 40, 41, 42, 43, 44, 45, 46, 47, 48, 49, 50, 51, 52, 53, 54, 55, 56, 57]. TAS2Rs are now recognized to play a critical role in immune defense by detecting bacterial by products, triggering immune responses, and modulating inflammatory pathways.

**Table 1:** Expression profiles of human bitter taste receptors (TAS2Rs) across various anatomic locations and cell types.

Anatomic location	Cell type	Receptors expressed	Reference
Oral cavity	Gingival epithelial cells	TAS2R14 TAS2R38	[10, 11]
	Gingival fibroblasts	TAS2R16 TAS2R31 TAS2R38 TAS2R39 TAS2R43 TAS2R50	[12, 13, 14, 15]
	Dental pulp stem cells	TAS2R10 TAS2R14 TAS2R19 TAS2R30 TAS2R31	[16]
Respiratory tract	Sinonasal epithelial cells	TAS2R4 TAS2R16 TAS2R38 TAS2R46 TAS2R47	[17, 18, 19]
	Airway smooth muscle	TAS2R1 TAS2R3 TAS2R4 TAS2R5 TAS2R8 TAS2R9 TAS2R10 TAS2R13 TAS2R14 TAS2R19 TAS2R20 TAS2R30 TAS2R31 TAS2R42 TAS2R45 TAS2R46 TAS2R50	[20, 21]
	Bronchial epithelial cells	TAS2R3 TAS2R4 TAS2R5 TAS2R7 TAS2R8 TAS2R9 TAS2R10 TAS2R13 TAS2R14 TAS2R19 TAS2R20 TAS2R31 TAS2R38 TAS2R39 TAS2R43 TAS2R45 TAS2R46 TAS2R50	[22]
	Ciliated cells	TAS2R4 TAS2R14 TAS2R16 TAS2R38 TAS2R43 TAS2R46	[23, 24]
Urogenital tract	Testis whole tissue	TAS2R3 TAS2R4 TAS2R14 TAS2R19 TAS2R43	[25]
	Spermatozoa	TAS2R3 TAS2R4 TAS2R14 TAS2R19 TAS2R43	[25, 26]
	Prostate epithelial cells	TAS2R1 TAS2R4 TAS2R10 TAS2R14 TAS2R38	[27]
	Ovarian epithelial cells	TAS2R1 TAS2R4 TAS2R10 TAS2R14	[27]
	Granulosa cells	TAS2R3 TAS2R4 TAS2R14 TAS2R19 TAS2R31 TAS2R43	[28, 29, 30]
	Uterine smooth muscle cells	TAS2R4 TAS2R5 TAS2R7 TAS2R8 TAS2R10 TAS2R13 TAS2R14 TAS2R31 TAS2R39 TAS2R42 TAS2R43 TAS2R45 TAS2R50	[31, 32]
	Placental epithelial cells	TAS2R14 TAS2R38	[33, 34]
	Detrusor smooth muscle cells	TAS2R1 TAS2R4 TAS2R5 TAS2R7 TAS2R8 TAS2R9 TAS2R10 TAS2R13 TAS2R14 TAS2R20 TAS2R30 TAS2R31 TAS2R38 TAS2R39 TAS2R40 TAS2R45 TAS2R50 TAS2R60	[35, 36]
Gastrointestinal tract	Stomach whole tissue	TAS2R4 TAS2R5 TAS2R10 TAS2R14 TAS2R19 TAS2R20 TAS2R31	[37, 38]
	Gastric parietal cells	TAS2R1 TAS2R3 TAS2R4 TAS2R5 TAS2R7 TAS2R9 TAS2R10 TAS2R13 TAS2R14 TAS2R16 TAS2R19 TAS2R20 TAS2R30 TAS2R31 TAS2R38 TAS2R39 TAS2R40 TAS2R41 TAS2R42 TAS2R43 TAS2R46 TAS2R50	[39, 40]
	Duodenum whole tissue	TAS2R4 TAS2R5 TAS2R14 TAS2R20 TAS2R38	[37]
	Jejunum whole tissue	TAS2R3 TAS2R4 TAS2R5 TAS2R7 TAS2R8 TAS2R10 TAS2R13 TAS2R14 TAS2R19 TAS2R20 TAS2R30 TAS2R31 TAS2R38 TAS2R39 TAS2R40 TAS2R43 TAS2R46	[37, 41]
	Ileum whole tissue	TAS2R3 TAS2R4 TAS2R5 TAS2R10 TAS2R14 TAS2R19 TAS2R20 TAS2R31 TAS2R38 TAS2R43	[37]
	Colon whole tissue	TAS2R1 TAS2R3 TAS2R4 TAS2R5 TAS2R10 TAS2R13 TAS2R14 TAS2R19 TAS2R20 TAS2R30 TAS2R31 TAS2R38 TAS2R39 TAS2R40 TAS2R42 TAS2R43 TAS2R45 TAS2R46 TAS2R50 TAS2R60	[37]
	Colonic enteroendocrine cells	TAS2R38	[42]
	Esophagus whole tissue	TAS2R4 TAS2R5 TAS2R10 TAS2R14 TAS2R19 TAS2R20 TAS2R31	[37]
	Liver whole tissue	TAS2R4 TAS2R5 TAS2R10 TAS2R13 TAS2R14 TAS2R19 TAS2R20 TAS2R30 TAS2R31 TAS2R43 TAS2R46	[43]
	Pancreatic islet cells	TAS2R3 TAS2R4 TAS2R5 TAS2R9 TAS2R10 TAS2R13 TAS2R14 TAS2R19 TAS2R20 TAS2R31 TAS2R43 TAS2R45 TAS2R46 TAS2R50 TAS2R60	[43]
Immune cells	Mast cells	TAS2R3 TAS2R4 TAS2R5 TAS2R10 TAS2R13 TAS2R14 TAS2R19 TAS2R20 TAS2R46	[44]
	Neutrophils	TAS2R4 TAS2R5 TAS2R10 TAS2R13 TAS2R14 TAS2R19 TAS2R20 TAS2R31 TAS2R38 TAS2R45 TAS2R46 TAS2R50	[45]
	Macrophages	TAS2R3 TAS2R4 TAS2R5 TAS2R7 TAS2R8 TAS2R9 TAS2R10 TAS2R14 TAS2R19 TAS2R20 TAS2R30 TAS2R31 TAS2R38 TAS2R39 TAS2R43 TAS2R45 TAS2R46	[46, 47]
	Lymphocytes	TAS2R4 TAS2R5 TAS2R10 TAS2R13 TAS2R14 TAS2R19 TAS2R20 TAS2R31 TAS2R38 TAS2R45 TAS2R46 TAS2R50	[45, 48]
Skin	Keratinocytes	TAS2R1 TAS2R3 TAS2R4 TAS2R5 TAS2R7 TAS2R8 TAS2R9 TAS2R10 TAS2R13 TAS2R14 TAS2R16 TAS2R19 TAS2R20 TAS2R30 TAS2R31 TAS2R38 TAS2R39 TAS2R40 TAS2R41 TAS2R42 TAS2R43 TAS2R45 TAS2R46 TAS2R50 TAS2R60	[49, 50, 51]
Cardiovascular system	Heart whole tissue	TAS2R3 TAS2R4 TAS2R5 TAS2R9 TAS2R10 TAS2R13 TAS2R14 TAS2R19 TAS2R20 TAS2R30 TAS2R31 TAS2R39 TAS2R43 TAS2R45 TAS2R46 TAS2R50	[52, 53]
	Vascular smooth muscle cells	TAS2R1 TAS2R3 TAS2R4 TAS2R5 TAS2R7 TAS2R9 TAS2R10 TAS2R13 TAS2R14 TAS2R42 TAS2R43 TAS2R44 TAS2R45 TAS2R46 TAS2R47 TAS2R48 TAS2R49 TAS2R50 TAS2R60	[54, 55, 56]
	Endothelial cells	TAS2R1 TAS2R3 TAS2R4 TAS2R5 TAS2R7 TAS2R8 TAS2R9 TAS2R10 TAS2R13 TAS2R14 TAS2R16 TAS2R19 TAS2R20 TAS2R30 TAS2R31 TAS2R38 TAS2R39 TAS2R40 TAS2R41 TAS2R42 TAS2R43 TAS2R45 TAS2R46 TAS2R50 TAS2R60	[57]

*Note:* This table illustrates the distribution and expression of bitter taste receptors (TAS2Rs) across different human tissues and cell types.

### 1.2 TAS2R Polymorphisms

Researchers have identified several polymorphisms in the TAS2R gene family that may account for differences in taste preference. A robust study was conducted by Wooding and Ramirez in 2022, which involved whole‐genome sequencing of 2500 subjects spanning the globe [58]. They identified 721 single‐nucleotide polymorphisms (SNPs), 9 short insertion‐deletion polymorphisms, and 8 large structural variants [58]. Among the identified SNPs, 525 were nonsynonymous substitutions and 196 were synonymous [58]. These variations could affect susceptibility to infection, highlighting the potential role of TAS2Rs in immunity. The most common polymorphisms studied to date are expressed in Table 2 [59, 60, 61, 62].

**Table 2:** Genetic polymorphisms in human bitter taste receptors.

Gene	Haplotype/genotype	Key polymorphism	Phenotype	Reference
TAS2R38	PAV/PAV	Pro‐Ala‐Val	Supertaster	[59]
	PAV/AVI	Pro‐Ala‐Val/Ala‐Val‐Ile	Medium taster	[59]
	AVI/AVI	Ala‐Val‐Ile	Nontaster	[59]
TAS2R43	W35W	Tryptophan at position 35	Hypersensitive	[60]
	W35S	Serine at position 35	Reduced function	[60]
TAS2R16	K172N	Asparagine	Increased sensitivity	[61]
TAS2R31	A227A	Alanine	Normal taster	[62]
	A227V	Valine	Variable	[62]

*Note:* This table summarizes key haplotypes/genotypes and their phenotypic classifications associated with bitter taste sensitivity levels.

### 1.3 TAS2Rs Role in Maintaining Homeostasis

The diets of humans, their ancestors, and their primate relatives have been predominantly plant‐based for millions of years [63]. Plants offer an accessible and nutritionally valuable food source, but they have also evolved numerous defense mechanisms to deter herbivory [64]. Chemical defenses play a crucial role in plant survival, as they produce an array of bitter‐tasting or toxic secondary metabolites, such as alkaloids, tannins, terpenoids, and cyanogenic glycosides, which function as deterrents by making consumption unpalatable or physiologically harmful to consumers [64]. These plant‐derived toxic compounds serve as ligands for TAS2Rs, which evolved as a key sensory system to detect and avoid potentially hazardous substances [65, 66]. The perception of bitterness provided a selective advantage by helping organisms avoid dangerous phytochemicals while still reaping the benefits of bitter compounds with medicinal or nutritional value. Beyond their role in taste perception, TAS2Rs are expressed in extraoral tissues and immune cells (Table 1), suggesting an expanded evolutionary function in homeostasis and systemic defense mechanisms. However, it is important to note that while the perception of bitterness is often associated with danger, it is not a definitive marker of toxicity [67]. Many bitter substances, such as caffeine and glucosinolates are not harmful and may even confer health benefits [67, 68]. Contrary, not all toxic compounds are bitter, some being tasteless or even pleasant in flavor [67]. This complexity reflects the evolutionary challenge of developing a sensory system that is sensitive enough to detect harmful agents without causing organisms to reject all bitter‐tasting substances. The evolutionary history of TAS2Rs highlights their role as early warning systems, enabling organisms to sense and respond to hazardous environments, increasing reproductive fitness and survival.

TAS2Rs exhibit both conservation and diversity across species. Among human TAS2Rs, amino acid sequence identities range from 23% to 86% [69, 70, 71]. Highly conserved regions suggest critical structural or functional roles, particularly in ligand‐binding pockets and intracellular signaling domains, ensuring proper bitter compound detection and downstream signaling via G proteins. These conserved features are common among GPCRs, maintaining receptor functionality across species. Conversely, lower sequence similarity may reflect neutral variation or changes that generate diversity in ligand specificity and sensitivity. In other species, such as mice, TAS2Rs show even lower sequence identity, ranging from 15% to 54% [72]. The mice repertoire includes more functional genes and fewer pseudogenes than in humans, reflecting species‐species evolutionary pressures [72]. This pattern of diversification is exhibited in other species as well. For example, frogs are estimated to have around 100 TAS2Rs [73, 74, 75], more than humans (26) or mice (~40) [74, 76], likely due to their need to detect diverse environmental toxins [77]. TAS2Rs balance evolutionary conservation for core functions with diversity for species‐specific needs, offering insights into toxin avoidance, immune regulation, and potential therapeutic targets.

### 1.4 TAS2Rs Function in *P. Aeruginosa* Respiratory Infection

As previously mentioned, the localization of TAS2Rs at barrier tissues throughout the body hints at a potential role beyond toxin avoidance in host defense. One pathogen that TAS2Rs have recently been implicated in responding to is *Pseudomonas aeruginosa. P. aeruginosa* is a ubiquitous organism with a propensity to inhabit a diverse array of environments, including soil, water, sewage, and hospital settings [78]. As a gram‐negative opportunistic pathogen, *P. aeruginosa* is a leading cause of nosocomial infections, often in immunocompromised patients [79, 80, 81, 82, 83]. *P. aeruginosa* establishes dominance in the host through quorum sensing, a cell‐density dependent mechanism governing biofilm formation, virulence and antibiotic resistance [79, 84, 85, 86]. *P. aeruginosa las* and *rhl* quorum sensing systems rely on diffuse acyl‐homoserine lactone (AHL) signaling molecules, *N*‐3(‐oxo‐dodecanoyl)‐L‐homoserine lactone (3‐oxo‐C_12_‐HSL) and *N*‐butanoyl‐L‐homoserine lactone (C_4_‐HSL) [87, 88, 89, 90]. A third system depends on the quinolones 2‐heptyl‐3‐hydroxy‐4‐quinolone (Pseudomonas quinolone signal, PQS) and 4‐hydroxy‐2‐heptyquinolone (HHQ) [91, 92, 93, 94]. Of particular concern is the ability of *P. aeruginosa* to chronically colonize the respiratory tract of individuals with chronic obstructive pulmonary disease, cystic fibrosis (CF), and chronic rhinosinusitis (CRS) [95, 96]. *P. aeruginosa's* proficiency for biofilm formation and hypermutability enables heightened antibiotic resistance, rendering current therapeutics obsolete [95].

**Figure 2:**
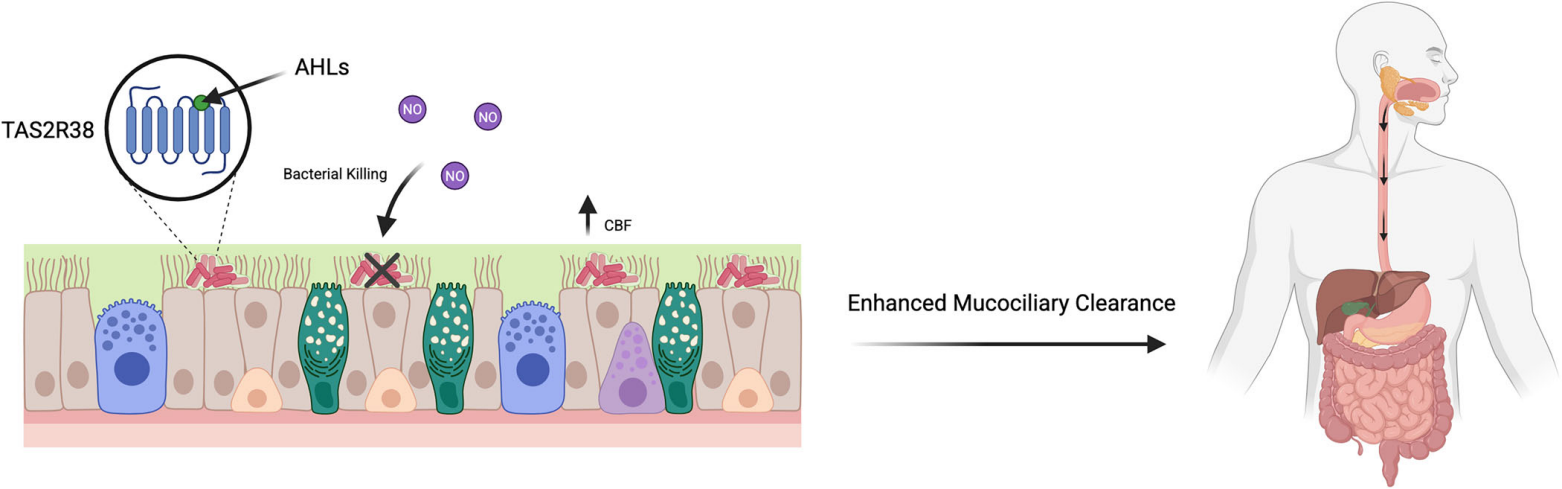
TAS2R38 detects *P. aeruginosa* acyl‐homoserine lactones (AHLs) in the airway to trigger mucociliary clearance. *P. aeruginosa* AHLs bind to TAS2R38 in the upper respiratory tract, resulting in nitric oxide (NO) production. NO diffuses into the air‐liquid interface (ALI), where it has direct bactericidal effects against *P. aeruginosa*. TAS2R38 triggers increased ciliary beating frequency (CBF) and enhanced mucociliary clearance, mediated by protein kinase G. The figure was created with Biorender.

It was recently discovered that human bitter taste receptors TAS2R4, 14, 16, and 38 expressed in the cilia of human sinonasal and bronchial epithelial cells detect *P. aeruginosa* QSMs [17, 23, 97]. Exploiting the host's ability to detect *P. aeruginosa* could have a profound impact on available treatment options. Lee et al. [17] reported that AHLs 3‐oxo‐C_12_‐HSL and C_4_‐HSL activate TAS2R38 expressed in human sinonasal epithelial cells (Figure 2). TAS2R38 activation results in Ca^2+^ release that culminates in endothelial nitric oxide synthase (eNOS) mediated NO production [17, 98]. The release of NO results in increased ciliary beat frequency (CBF) and enhanced mucociliary clearance, dependent on protein kinase G [17, 99]. Mucociliary clearance is essential for pathogen immobilization and transport to the oropharynx for subsequent expectoration [100, 101]. Importantly, NO diffuses into the air‐liquid interface (ALI) and has direct bactericidal effects against the PAO1 strain of *P. aeruginosa* [17, 98]. This study utilized a heterologous expression system to express TAS2R38 in HEK293 cells coupled to G_α16‐gust44_ to visualize calcium mobilization [17]. They further cultured human sinonasal and bronchial epithelial cells at the ALI [17]. However, further research is needed to assess the translatability of the results due to the lack of an in vivo model.

It was subsequently discovered that PQS is an agonist of TAS2R4, 16, and to a lesser extent 38, while HHQ is an agonist of TAS2R14 [99]. This redundancy in ligand recognition ensures multiple modes of signaling disruption, potentially providing increased protection against the spread of *P. aeruginosa*. In humans and mice, bitter taste signaling in sinonasal ciliated epithelial cells requires the TRPM5 ion channel and PLCβ2, but not the canonical taste G‐protein, α‐gustducin [17, 98, 102]. Conversely, it was discovered that non‐ciliated cells, known as solitary chemosensory cells (SCCs) in mice express TAS2Rs that are activated by AHLs in an α‐gustducin‐dependent manner. In this context, SCCs control breathing to prevent harmful substances from entering the airway [103, 104, 105].

**Figure 3:**
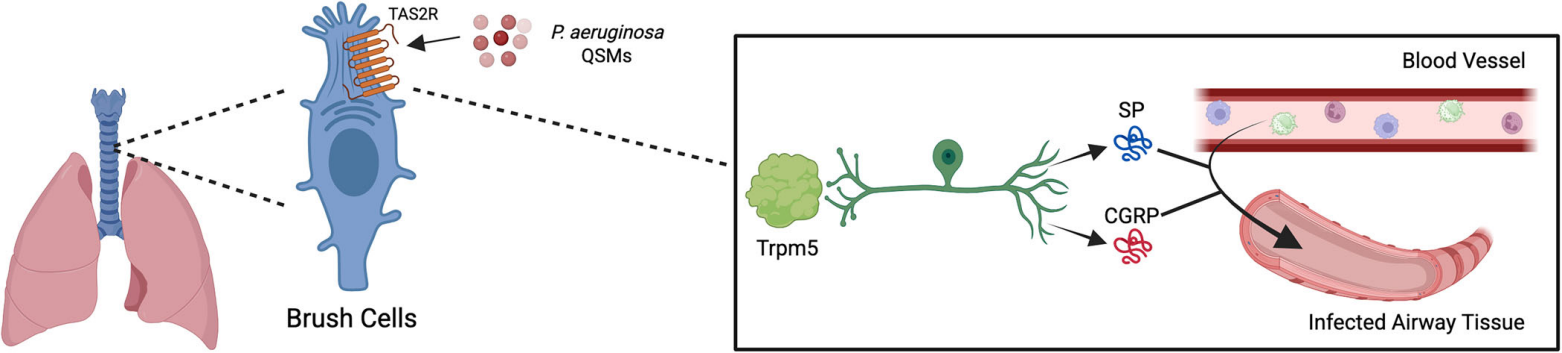
*P. aeruginosa* quorum sensing molecules (QSMs) activate TAS2Rs in tracheal brush cells to initiate an immune response. This activation results in the release of the neuropeptides substance P (SP) and calcitonin gene‐related peptide (CGRP) from sensory neurons, resulting in leukocyte recruitment from blood vessels to the site of infection. The figure was created with Biorender.

In the lower airway, TAS2Rs are expressed in a subtype of epithelial cell termed tracheal brush cells, which respond to QSMs produced by *P. aeruginosa* [106, 107, 108, 109, 110]. In mice, it was reported that bitter taste signaling in tracheal brush cells results in neurogenic inflammation dependent on cholinergic stimulation (Figure 3) [106]. TRPM5‐dependent signaling triggers the release of the neuropeptides CGRP (calcitonin gene‐related peptide) and substance P (SP) from sensory neurons, respectively [106]. CGRP and SP trigger an innate immune response by inducing plasma extravasation and neutrophil diapedesis [106]. Airway infection with *P. aeruginosa* CF isolate NH57388A led to the recruitment of neutrophils, monocytes, and NK cells to the bronchoalveolar lavage fluid 4 h post‐infection [106]. These changes were abrogated in Trpm5‐deficient mice [106]. This study was strengthened by the use of an in vivo model and a thorough investigation into the mediators released and downstream immune response. In a related study, PQS was shown to increase particle transport speed in isolated mouse tracheae by activating brush cells in a TRPM5‐dependent manner [107]. This is believed to be mediated by the release of acetylcholine, which acts in a paracrine manner on neighboring ciliated cells by binding to the M3R receptor [107]. Trpm5^−/−^ mice still possessed a residual response to PQS, and RNA‐seq detected the expression of Tas2Rs in ciliated cells [107]. Therefore, further studies are needed to isolate the effects of brush cell signaling in response to PQS. Furthermore, in vivo studies are required to verify whether this pathway is able to mount a significant immune response in a living organism.

TAS2Rs are expressed in neutrophils and macrophages, which migrate to the lung during bacterial infection [111]. It was discovered that Tas2r138, the mouse ortholog of human TAS2R38, led to increases in neutrophils and to a lesser extent in alveolar macrophages following *P. aeruginosa* lung infection or stimulation with 3‐oxo‐C_12_‐HSL [111]. Tas2r138 localizes in lipid droplets within neutrophils and competitively binds to the peroxisome proliferator‐activated receptor gamma (PPARγ) antagonist, 3‐oxo‐C_12_‐HSL [111, 112]. The release of 3‐oxo‐C_12_‐HSL from PPARγ allows PPARγ to translocate from the nucleus to the cytoplasm [111]. In the cytoplasm, PPARγ expedites lysosomal lipid droplet degradation by binding to perilipin‐2, a protein that mediates entry into the lysosome [111, 113]. Lipid droplet degradation facilitates the destruction of 3‐oxo‐C_12_‐HSL and therefore, interferes with bacterial quorum sensing and dispersal [111]. Consequently, the possession of TAS2R38 in neutrophils likely helps to contain *P. aeruginosa* infection, preventing spread into the bloodstream and downstream tissues. This is especially significant if initial TAS2R38 signaling in brush cells or sinonasal cells fails to mitigate the infection.

Overall, these studies indicate that TAS2Rs act as sensors for bacterial‐derived QSMs in epithelial and innate immune cells of the respiratory tract. However, few conclusions can be drawn regarding their role in human infection due to the lack of in vivo research. Future studies will require murine models to investigate the functionality and variation in TAS2R function during respiratory infection.

### 1.5 TAS2Rs in Cystic Fibrosis Lung Infection

CF is an autosomal recessive disease arising from approximately 700 mutations in the *cftr* gene that encodes the CF transmembrane conductance regulator (CFTR) [114]. The disease impairs the body's ability to efficiently regulate the movement of chloride, sodium, and water [115, 116, 117, 118]. This results in thickened mucus and inadequate mucociliary clearance, rendering the host more susceptible to airway pathogens, including *P. aeruginosa, Staphylococcus aureus, Aspergillus fumigatus*, and *Candida albicans* [114].

In a recent study by Singh et al. [114], primary human bronchial epithelial (HBE) cell lines from CF (BCF) and non‐CF (BD) donors, and immortalized HBE cell lines from CF (CuFi‐1) and non‐CF (NuLi‐1, BEAS‐2B) donors were studied to examine the role of TAS2R14 in CF respiratory infection [114]. They found significantly greater TAS2R14 mRNA expression in BCF compared to BD cells, but this did not translate to protein expression [114]. Treating BD and BCF cells with 3‐oxo‐C_12_‐HSL led to significant calcium mobilization that was abrogated in TAS2R14‐knockdown (KD) cells, demonstrating a TAS2R14‐dependent mechanism [114]. In addition, 3‐oxo‐C_12_‐HSL treatment resulted in significantly greater nitrate release in scramble shRNA‐treated CF HBE cells cultured at the ALI compared to TAS2R14 KD cells [114]. However, no difference was observed between CF and non‐CF cell lines [114]. On the contrary, BCF cells exhibited greater production of several cytokines in a TAS2R14‐dependent manner compared to BD cells [114]. This was coupled with upregulated G_αi_ expression and reduced cAMP production, potentially indicating enhanced signaling in response to TAS2R14 activation in CF patients [114]. However, it was unclear from this study how G_αi_ upregulation translates to enhanced cytokine release without impacting nitrate levels. Elucidating the signaling pathway upstream of cytokine release would have improved the clinical applicability and robustness of the findings. Furthermore, it was overlooked whether the cytokines confer a protective role through immune cell recruitment or if they promote excessive inflammation that damages the respiratory tissue.

In an earlier study, non‐CF primary nasal epithelial cells and CF cells from patients homozygous for the phenylalanine 508 deletion rendering CFTR non‐functional were cultured at the ALI [118]. No difference was detected in the expression of TAS2R4, 14, 16, and 38 or calcium mobilization in response to 3‐oxo‐C_12_‐HSL [118]. Strikingly, CF ALI cultures released significantly less NO in response to 3‐oxo‐C_12_‐HSL stimulation [118]. This translated to reduced CBF and impaired bacterial killing of five *P. aeruginosa* strains, including the lab strain PAO1 and clinical strain P11006 [118]. Although this is an exciting finding, conclusions are severely limited by the lack of an in vivo model. ALI models do not recapitulate the complex cell‐cell interactions with non‐epithelial cells and cell‐ECM matrix interactions, and they differ in stiffness from in vivo tissue [119]. It is also intriguing that NO levels differed between CF and non‐CF cells in nasal epithelial cells but not in HBE. This may indicate that TAS2Rs in nasal epithelial cells play a more significant role in the initial response to bacterial pathogens invading the airways.

To conclude, these studies reveal differential findings between CF bronchial and nasal epithelial cells. While CF bronchial epithelial cells produced greater cytokines in a TAS2R14‐dependent manner compared to non‐CF cells, CF nasal epithelial cells exhibited an impaired antibacterial response. Subsequent studies should further validate and investigate this variation to understand how TAS2R function is affected by dysfunctional CFTR.

### 1.6 TAS2Rs in Oral Bacterial Infection

The oral cavity harbors one of the most extensively characterized microbiomes, with 392 documented taxa, each represented by at least one reference genome, and nearly 1500 genomes sequenced to date [120]. The oral microbiome is essential for maintaining oral homeostasis, serving as a key regulator of local immune responses, protecting against pathogenic infections, and preventing dysbiosis‐linked conditions such as dental caries and periodontal disease. *Streptococcus mutans*, *Porphyromonas gingivalis*, and *Fusobacterium nucleatum* are among the most studied oral bacteria due to their roles in dysbiosis, driving dental caries, chronic periodontitis, and biofilm pathogenesis.

*S. mutans* produces three QSMs termed competence stimulating peptides (CSP), including CSP‐1, CSP‐2, and CSP‐3 [121]. CSP‐1 has been shown to activate TAS2R14 expressed in gingival epithelial cells (GECs), inducing Ca^2+^ mobilization dependent on the canonical bitter taste signaling pathway components, Gβγ and PLCβ, as well as autophagy component ATG‐7 [121]. CSP‐1 promoted neutrophil chemoattraction and the release of interleukin (IL)‐6, IL‐8, and tumor necrosis factor‐alpha (TNF‐α) from GECs [121]. However, this study by Medapati et al. [121] lacks in vivo validation and relies entirely on in vitro cell culture models, leaving open whether TAS2R14‐mediated responses actually influence *S. mutans* infection or caries development in a living system. Further investigation into this could include infecting TAS2R14 or TAS2R cluster knockout mice with *S. mutans* to assess changes in bacterial colonization, biofilm formation, enamel demineralization and oral mucosal immune responses. In vivo models reveal confounding factors not captured by cell line studies. For example, mast cells, which are abundant in the oral cavity, express the receptor MRGPRX2, which can also bind CSP‐1 [122, 123]. This interaction, absent in cell line experiments, may influence the results observed in the paper when transitioning to an in vivo model. As well, the persistence of NF‐κB activation after TAS2R14 knockdown suggests compensatory pathways. While the TAS2R14 knockdown experiments demonstrate that this receptor is necessary for CSP‐1‐induced calcium signaling in GECs, they do not conclusively prove that CSP‐1 directly binds to TAS2R14. Several alternative explanations remain plausible, such as binding another receptor in a complex with TAS2R14, triggering secondary signals that activate TAS2R14, or through off‐target siRNA effects. Direct binding studies are needed to confirm whether CSP‐1 is a true TAS2R14 ligand.

Another study conducted by Medapati et al. [10] demonstrates that TAS2R14‐expressing GECs play a critical role in the internalization and immune response against *Staphylococcus aureus*. Knockdown of TAS2R14 significantly reduces *S. aureus* uptake, which is dependent on F‐actin polymerization, as inhibition with cytochalasin D abolishes internalization [10]. TAS2R14 likely promotes cytoskeletal reorganization by regulating Rac1/PAK1 signaling, leading to actin remodeling necessary for bacterial uptake [10]. Furthermore, TAS2R14‐expressing GECs secrete higher levels of the antimicrobial peptide human beta‐defensin‐2 (hBD‐2) in response to *S. aureus*, whereas TAS2R14‐deficient cells fail to mount this defense [10]. Conditioned media from TAS2R14‐expressing GECs also inhibit *S. aureus* growth, suggesting that TAS2R14 activation triggers bactericidal factors [10]. These findings highlight TAS2R14 as a key mediator of innate immune responses against *S. aureus*, influencing bacterial internalization, cytoskeletal dynamics, and antimicrobial defense mechanisms in oral epithelial cells. This study also investigated *S. mutans* and found that TAS2R14 knockdown does not affect *S. mutans* internalization or hBD‐2 secretion; however, this does not explain why [10]. This study provides compelling evidence that TAS2R14 plays a key role in GEC defenses against *S. aureus*; however, the findings raise several critical questions. First, the mechanistic link between TAS2R14 and bacterial sensing remains unclear—does *S. aureus* directly activate TAS2R14 via secreted metabolites, or is the receptor responding to host‐derived signals during infection? Second, the striking lack of TAS2R14‐dependent responses to *S. mutans* suggests pathogen‐specific evasion mechanisms, possibly mediated by biofilms, which were not explored. Future work should test whether *S. mutans* biofilms physically shield bacterial ligands from TAS2R14 or actively suppress receptor signaling. Finally, the study's reliance on in vitro models limits translational relevance—validation in animal models would clarify its role in oral infection.

Gil et al. [11] show that another TAS2R, TAS2R38, plays an important role within the oral cavity. Particularly, they demonstrate that the PAV genotype (Table 2) of TAS2R38 provides robust protection against *S. mutans* through multiple synergistic mechanisms. PAV/PAV carriers showed a 4.3‐fold upregulation of TAS2R38 expression when exposed to *S. mutans*, triggering a potent hBD‐2 antimicrobial response that was 77% dependent on TAS2R38 signaling [11]. This receptor‐mediated defense was further amplified by increased secretion of IL‐1α, suggesting that TAS2R38 coordinates both direct antimicrobial activity and inflammatory responses against cariogenic bacteria [11]. Importantly, this protection appears specific to *S. mutans* planktonic forms, as biofilms may evade detection—a phenomenon observed with other taste receptors like TAS2R14 [11]. However, TAS2R38's role extends beyond cariogenic bacteria. Several studies have also shown that children with the non‐taster polymorphism had higher *S. mutans* counts, as well as more severe caries than those with the taster polymorphism, likely due to increased sugar intake promoting bacterial growth [124, 125]. The study by Gil et al. [11] reveals a genotype‐dependent immune polarization, where AVI/AVI (Table 2) carriers mounted stronger responses to periodontal pathogens such as *Porphyromonas gingivalis*. When challenged with *P. gingivalis*, AVI/AVI GECs showed a 4.4‐fold increase in TAS2R38 expression and elevated secretion of both IL‐1α and IL‐8, suggesting enhanced defenses against periodontal infection [11]. Meanwhile, *Fusobacterium nucleatum*, another periodontal pathogen, elicited weaker overall TAS2R38 activation but triggered compensatory hBD‐2 release in AVI/AVI cells upon receptor knockdown, indicating pathogen‐specific adaptation of immune strategies [11].

Further investigation into TAS2R38 polymorphisms by Mei et al. [126] explores the link between genetic variations in the bitter taste receptor gene TAS2R38, oral bacteria, and halitosis. Individuals with the AVI/AVI genotype were found to have a higher prevalence of halitosis and poorer treatment outcomes compared to those with functional variants [126]. This is because TAS2R38 not only affects taste perception but also plays a role in immune defense against oral bacteria [126]. The study found that AVI/AVI carriers had higher levels of *Prevotella intermedia*, a bacterium that produces foul‐smelling sulphur compounds, and even after treatment, harmful bacteria like *Tannerella, Filifactor*, and *Mycoplasma* persisted in these individuals, explaining their poorer response to therapy [126]. Functional variants of the gene enhance immune responses like mucociliary clearance and antimicrobial activity, but AVI/AVI individuals lack this defense, allowing odor‐causing bacteria to thrive [126]. While the study focused on the salivary microbiome and had limitations like sample size, it suggests that TAS2Rs could help predict halitosis risk and treatment success.

In mice, gingival SCCs express Tas2rs and signaling components of TAS2Rs, such as α‐gustducin (Gnat3), Trpm5, and PLCβ2, which help regulate oral microbiota and prevent periodontitis [127]. Gnat3‐deficient mice exhibit altered microbiota, increased alveolar bone loss, and more severe periodontitis, with an overgrowth of pathogenic *Pasteurella*, a pathogen associated with periodontitis [127]. Activating Tas2r105 with denatonium benzoate in wild‐type mice was shown to upregulate antimicrobial β‐defensin‐3, reducing bone loss and bacterial load—an effect absent in Gnat3^−/−^ mice, confirming the pathway's role, and thus the importance of Tas2r105 activation in regards to periodontitis [127]. Similarly, primary human gingival fibroblasts express 19 distinct TAS2Rs along with key signaling components—α‐gustducin, PLCβ2, and TRPM4, but notably not TRPM5 [128]. During periodontitis, these fibroblasts respond to bacterial pathogen‐associated molecular patterns, such as lipopolysaccharide (LPS), by releasing pro‐inflammatory cytokines that drive disease progression [128]. However, when stimulated simultaneously with LPS and the TAS2R16 agonist salicin, they exhibit a marked reduction in inflammatory mediators, including IL‐6, IL‐8, and CXCL3, as well as decreased neutrophil recruitment [128]. This anti‐inflammatory effect is mediated through TAS2R16‐dependent suppression of intracellular cAMP and inhibition of NF‐κB signaling [128].

Together, these findings highlight the role of TAS2Rs in maintaining oral microbial homeostasis and modulating host‐pathogen interactions, where specific receptors like TAS2R14 and TAS2R38 differentially respond to cariogenic and periodontal pathogens through distinct antimicrobial and inflammatory pathways. While TAS2R activation shows promise in mitigating oral dysbiosis, key gaps remain in understanding how biofilm evasion and receptor polymorphisms shape clinical outcomes. Future studies should prioritize in vivo models of caries and periodontitis to evaluate whether pharmacologically targeting these receptors can restore microbial balance or delay oral disease progression.

### 1.7 TAS2Rs in Intestinal Infection

**Figure 4:**
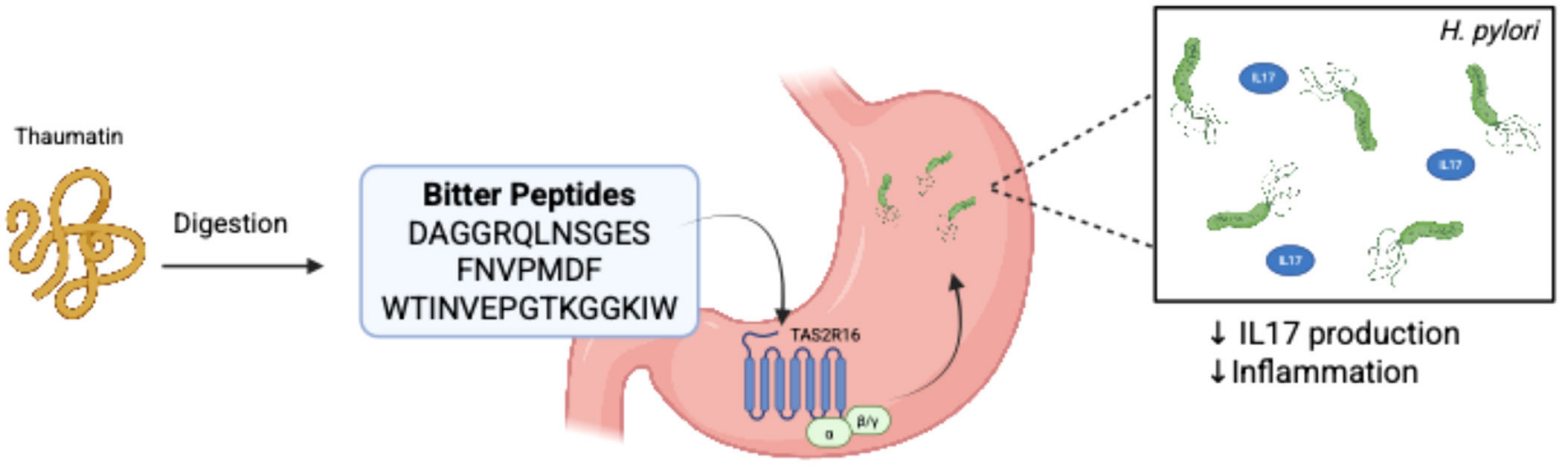
Thaumatin‐derived bitter peptides modulate gastric immunity via TAS2R16 activation. Upon gastric digestion, the sweet‐tasting protein thaumatin is broken down into bitter peptides (DAGGRQLNSGES, FNVPMDF, WTINVEPGTKGGKIW), which are detected at physiologically relevant concentrations. These peptides activate the bitter taste receptor TAS2R16 on gastric parietal HGT‐1 cells, triggering proton secretion and downregulating *Helicobacter pylori*‐induced IL‐17 production. The figure was created with Biorender.

Recent research has demonstrated that the sweet‐tasting protein thaumatin undergoes gastric digestion to release bitter peptides, which modulate bitter taste receptor‐mediated responses in the stomach [39]. In vitro and in vivo porcine digestion studies identified key bitter peptides—DAGGRQLNSGES, FNVPMDF, and WTINVEPGTKGGKIW—whose sensory bitterness was confirmed [39]. These peptides, detectable at physiologically relevant nanomolar concentrations, were found to stimulate proton secretion in human parietal HGT‐1 cells via TAS2R16 [39]. Notably, the peptides significantly reduced *Helicobacter pylori*‐induced secretion of the pro‐inflammatory cytokine IL‐17 (Figure 4) [39]. This anti‐inflammatory effect was abolished upon TAS2R16 knockdown or inhibition with probenecid, confirming receptor dependence [39]. The findings reveal a novel mechanism by which thaumatin‐derived bitter peptides, generated during digestion, may exert protective effects against *H. pylori*‐driven inflammation, suggesting potential dietary implications for managing gastric inflammatory responses (Figure 4). This study excelled by integrating proteomics, sensory science, immunology, and molecular biology to comprehensively investigate how thaumatin‐derived peptides influence gastric immunity. This multidisciplinary framework strengthens the conclusion that dietary proteins can modulate innate immunity via bitter taste receptors—a novel concept with implications for both nutrition and gastroenterology. The use of pigs as an in vivo digestion model was well justified, given their gastro‐anatomical and enzymatic similarities to humans, including comparable gastric pH, pepsin activity, and transit times [39, 129]. By showing that 83.7% of peptides identified in pigs matched the in vitro digestion profile, the study bolstered the translational relevance of its findings [39]. Pigs also allowed for real‐time sampling of gastric contents, providing critical validation that thaumatin‐derived peptides reach physiologically detectable levels—a key consideration for future dietary interventions. However, the study's focus on IL‐17A reduction in isolated HGT‐1 cells, while insightful, presents an oversimplified view of gastric immune responses. In vivo, the anti‐inflammatory effects of thaumatin‐derived peptides would likely involve complex crosstalk with other immune cells such as macrophages and T cells, as well as modulation of additional cytokines like IL‐8 and TNF‐α that play critical roles in *H. pylori*‐induced inflammation [130]. Furthermore, while the acute anti‐inflammatory effects were clearly demonstrated, the long‐term consequences of regular thaumatin consumption remain uncertain. Chronic exposure to these bitter peptides could potentially lead to TAS2R desensitization or compensatory immune responses, factors that were not addressed in this study but could significantly impact the therapeutic potential of this approach. These limitations do not undermine the study's significance but highlight opportunities for future research—particularly in human trials and mechanistic studies of immune cell networks.

A study by Liszt et al. [60] reveals how TAS2Rs in intestinal epithelial cells critically regulate host defense against *Escherichia coli* through receptor‐specific mechanisms [60]. Notably, genetic polymorphisms in TAS2R43 create distinct phenotypic responses to bacterial challenges. Approximately 33% of the population carries a complete TAS2R43 gene deletion (TAS2R43−), rendering them insensitive to TAS2R43 agonists [60]. Among TAS2R43+ individuals, a crucial amino acid polymorphism at position 35 (tryptophan [W] vs. serine [S]) further determines receptor sensitivity (Table 2) [60]. The W variant confers high sensitivity to bitter compounds like aloin, while the S variant shows reduced responsiveness [60]. Activation of TAS2R10 by denatonium benzoate triggered Ca²⁺‐dependent release of antimicrobial peptides from Paneth cells, significantly suppressing *E. coli* growth—an effect amplified in obesity but absent in lean individuals [60]. Conversely, TAS2R43 stimulation produced genotype‐dependent effects: aloin enhanced *E. coli* proliferation in TAS2R43 + W individuals through goblet cell secretion of the mucolytic glycoprotein CLCA1, which served as a bacterial nutrient source, while TAS2R43 + S individuals showed weaker responses and TAS2R43− individuals were completely unaffected [60]. This reveals that the same bitter compound can produce opposite effects on *E. coli* growth depending entirely on the host's TAS2R43 genotype. The dichotomy in outcomes seen by inhibition via TAS2R10 versus genotype‐dependent promotion via TAS2R43 highlights the complex interplay between host genetics and microbial responses in the gut. This study makes contributions to our current understanding of TAS2Rs in the intestinal tract by elucidating how TAS2R10 and TAS2R43 differentially regulate gut defense against *E. coli* through antimicrobial peptide secretion and mucus remodeling, respectively, while revealing clinically important modulation by obesity and genetic polymorphisms. However, the work leaves critical signaling mechanisms unresolved. While demonstrating calcium flux, it fails to delineate the complete G‐protein coupling, secondary messengers, or downstream effectors that distinguish TAS2R10's antibacterial effects from TAS2R43's mucus‐modifying actions. The study also misses opportunities to connect these pathways to known gut defense systems, notably IL‐17/IL‐22 signaling and NOD2 crosstalk, that likely interact with bitter receptor activation [131].

Lei et al. [132] utilized a mouse model to investigate *Ruminococcus gnavus* infection, where oral gavage administration led to selective tuft cell expansion in the proximal colon but not in the small intestine or mid‐colon. This response was mediated by TAS2Rs, particularly Tas2r126 and Tas2r129, which were significantly upregulated following infection [132]. Pharmacological inhibition of TAS2Rs with allyl isothiocyanate attenuated IL‐25 release, while rectal administration of bitter compounds mimicked the infection‐induced tuft cell expansion, demonstrating the functional importance of these receptors [132]. Single‐cell RNA sequencing confirmed that the effects were TAS2R‐mediated rather than through alternative pathways like TRPA1 [132]. The findings reveal a chemosensory mechanism in which *R. gnavus* activates colonic TAS2Rs, triggering a Gγ13‐PLCβ2‐Trpm5‐IL‐25 signaling cascade that drives tuft cell hyperplasia while maintaining epithelial homeostasis through gasdermin‐mediated protection against apoptosis [132]. These results from mouse models provide important insights into host‐microbe interactions that may underlie *R. gnavus*‐associated gastrointestinal pathologies in humans. The study's demonstration of regional specificity in intestinal responses to *R. gnavus* infection reveals important insights into gut segmental physiology. This compartmentalization could explain why *R. gnavus* is associated with proximal‐predominant conditions like IBD, suggesting location‐specific host‐microbe crosstalk [133, 134]. However, the failure to identify the exact bacterial ligands activating TAS2Rs represents a significant knowledge gap. Without characterizing the specific molecular triggers, the translational potential remains limited.

Collectively, these studies highlight the emerging role of TAS2Rs as critical mediators of host‐microbe interactions in the gut, influencing immune responses, epithelial defense, and microbial colonization in a receptor‐ and region‐specific manner.

### 1.8 Clinical Relevance and Implications

As research continues to advance, it is becoming increasingly clear that TAS2Rs play a key role beyond taste perception, particularly in modulating immune responses and influencing susceptibility to infection. Emerging evidence suggests that genetic polymorphisms in TAS2Rs, specifically TAS2R38 and TAS2R43, can impact host‐pathogen interactions and immune function. This highlights the potential for TAS2R genotyping to be developed as a predictive biomarker in infectious disease contexts, with implications for both risk stratification and personalized therapeutic strategies.

For instance, the TAS2R38 receptor, particularly in individuals with the PAV/PAV genotype, has been shown to provide robust protection against *S. mutans*, a key driver of dental caries [11]. Conversely, AVI/AVI carriers, who have reduced TAS2R38 function, show stronger immune responses to pathogens like *P. gingivalis*, but also have an increased prevalence of halitosis, possibly due to altered microbial clearance [11]. Further expanding this idea, TAS2R43 polymorphisms have also been linked to host‐pathogen dynamics. In individuals with the TAS2R43 + W variant, exposure to aloin enhanced *E. coli* colonization [60]. In contrast, TAS2R43 + S individuals exhibited attenuated responses, and TAS2R43− individuals showed no effect at all, highlighting how genetic variation in bitter taste receptors may directly modulate immunity and pathogen behavior [60].

Variations in TAS2R38 genotype and phenotype also have potential implications for susceptibility to CRS and CF. Individuals with at least one non‐functional allele (PAV/AVI) or (AVI/AVI) have reduced TAS2R38 signaling in response to *P. aeruginosa* AHLs and are thus more susceptible to sinonasal respiratory infections [17]. However, the applicability in clinical settings is currently under debate. Several studies found that TAS2R38 genotype and phenotype correlate with the presence of CRS and CF, symptom severity, and the necessity or success of surgical intervention [135, 136, 137, 138, 139, 140, 141, 142, 143, 144]. For instance, Adappa et al. [139] discovered that variation in endoscopic sinus surgery outcomes can partly be explained by TAS2R38 polymorphisms. PAV/PAV patients who underwent sinus surgery for non‐polyploid CRS had a quality‐of‐life improvement of 38 ± 21 versus 12 ± 22 (*p* = 0.006) in non‐PAV/PAV patients at 6 months [139]. However, other studies did not find an association between TAS2R38 genotype and chronic lung infection [145, 146].

In an Italian population, there was no significant difference in the proportion of PAV/PAV, PAV/AVI, and AVI/AVI genotypes between controls and CRS patients or between CRS patients with or without nasal polyps [145]. Although differential results can partly be explained by sample size, CRS is a complex disease dependent on more than one genetic loci [145]. Differences in overall genetic makeup across populations could explain the discrepancies in the results. Moreover, TAS2R38 activation has only been investigated in vitro in response to gram‐negative AHLs [145]. CRS involves a much larger bacterial and fungal repertoire, so it remains unclear whether TAS2R38 plays a significant role in vivo [147].

Existing studies highlight the potential to modulate the immune response to infection by treating individuals with TAS2R‐specific agonists. To illustrate, diphenhydramine, an H1 antihistamine and agonist of TAS2R14, was administered to nasal ALI cultures [148]. Pharmacological treatment with diphenhydramine increased NO production and CBF [148]. However, pre‐treatment with *P. aeruginosa* flagellin, a TLR5 agonist, significantly reduced this enhancement by downregulating TAS2R14 expression [148, 149]. Diphenhydramine repressed the planktonic growth of *S. aureus* strain M2, PAO1, and *P*. aeruginosa clinical strains [148]. Interestingly, diphenhydramine was able to inhibit biofilm formation of PAO1 and *P. aeruginosa* clinical strain P11006, in addition to adherence to CF bronchial epithelial cells [148]. The results of this study demonstrate that TAS2R agonists such as diphenhydramine could be modified to enhance the host's response to infection by exploiting pre‐existing antibacterial immunity. However, the finding that flagellin decreases this enhancement demonstrates that in vivo follow‐up studies are crucial in determining how ligand or cell based interactions can affect results.

Together, these findings underscore the emerging potential of TAS2R genotyping not only as a biomarker of taste perception but also as a predictive tool for infection susceptibility and therapeutic responses. This precision‐medicine approach could ultimately reshape how we understand and manage host‐pathogen interactions in clinical practice.

## 2 Future Directions

### 2.1 Expanding TAS2R Research to Other Sites of Infection

Current research regarding TAS2Rs and bacterial infection is limited to the respiratory tract, gastrointestinal tract, and oral cavity. Based on Table 1, it is evident that TAS2Rs are found in a variety of other tissues, such as the skin and urogenital tract. These tissues are major portals of entry for bacteria due to their localization at the host‐environment interface. For instance, *S. aureus* commonly causes skin and soft tissue infections (SSTIs) and is frequently resistant to antibiotics [150]. From 2010 to 2020, the incidence of SSTIs in the US was 77.5 per 1000 person‐years of observation and often necessitated hospitalization [150]. Furthermore, antibiotic‐resistant *E. coli* is a major concern in urinary tract infections within the emergency department [151]. TAS2Rs wide tissue distribution and ability to recognize both gram‐positive and gram‐negative bacteria hint at their potential role in host defense under diverse physiological contexts that should be explored in more detail, especially due to the incidence and growing concern of antibiotic‐resistance.

### 2.2 Investigating How TAS2R Polymorphisms Impact the Hosts Response to Bacterial Infection

The study by Wooding & Ramirez uncovered an estimated 169 SNPs that are expected to alter TAS2R receptor function [58]. For many of these polymorphisms, a connection has yet to be established between genetic variation, phenotypic variation, and susceptibility or outcome of infection [58]. To illustrate, it is unclear whether TAS2R16 polymorphisms alter the oral microbiota through modulation of taste preferences or if certain variants encode non‐functional receptors with reduced immunomodulatory capacities [152]. For TAS2R38, the AVI/AVI genotype was shown to be more susceptible to bacterial infection in ALI cultures [17]. Results are more conflicting in human studies, suggesting a potential confounding factor. It would be interesting to investigate how differential dietary habits according to taster status impact susceptibility and outcome of infection.

### 2.3 Cryo‐Electron Microscopy to Elucidate TAS2R Structure and Ligand Binding Sites

Cryo‐electron microscopy (cryo‐EM) is a revolutionary technique that can be used to decipher receptor structure and ligand binding sites. Studies have recently employed cryo‐EM to investigate TAS2R14 and TAS2R46, uncovering critical insights into the properties of these receptors. Cryo‐EM has revealed that TAS2R14 couples with both G_gust_ and G_i1_ and contains a novel positive allosteric modulator (PAM)‐bound allosteric site for intracellular tastants in addition to an orthosteric binding pocket occupied by cholesterol [153, 154, 155]. Cholesterol is required for the basal activity of G_i1_ and G_gust_, and positively modulates responses to intracellular tastants [155]. Intriguingly, researchers identified a deep cavity that bridges the orthosteric pocket and allosteric site occupied by many aromatic residues, suggesting that a variety of sterols could occupy this orthosteric pocket [155]. The ability of TAS2R14 to detect both extracellular and intracellular compounds and the possession of numerous ligand binding sites likely explain its broad ligand specificity [153, 154]. Intriguingly, Xu et al. [156] discovered that TAS2R46 has several features which distinguish it from other GPCR‐G protein complexes, such as the arrangement of TM3‐TM4‐TM5. Future studies should implement cryo‐EM to decipher the mechanism through which individual QSMs bind to their target TAS2Rs. Elucidating this interaction will be critical for the design of targeted agonists or antagonists. However, this is complicated by the fact that we are still uncertain whether microbial or host‐derived molecules stimulate TAS2Rs for select bacteria.

### 2.4 In Vivo Study of Bitter Taste Receptors

Human and mouse bitter taste receptors possess a variety of dissimilarities, severely limiting our ability to study them in vivo. Humans possess 26 TAS2R gene isoforms while mice possess ~40 [74, 76]. This is further complicated by differences in sensitivity and significant ligand heterogeneity between human and mice orthologous pairs [65, 66]. The vast majority of studies have utilized HEK293T cells co‐transfected with the chimeric G protein G_α16‐gust44_, allowing Ca^2+^ to be released following receptor activation [157]. Under this expression system, individual TAS2Rs can be heterologously expressed to examine responses to distinct molecules. Although this method can be used to discern the agonists of individual bitter taste receptors, it does not allow us to paint a full picture. Agonists which activate TAS2Rs in vitro may have no or significantly reduced effects in vivo due to differential absorbance, receptor expression, tissue distribution, and half‐life.

Since numerous Tas2rs are broadly tuned and can be activated by more than one ligand, it would likely prove ineffective to knockout individual Tas2rs in mice [66, 158]. Alternatively, most of the Tas2rs could be simultaneously knocked out due to their tight clustering on chromosome 6 [66, 159]. Mouse knockout lines have been developed for some but not all Tas2rs, and future research should place more emphasis on designing these models to improve the applicability of findings. Various mouse lines have been developed which diminish bitter taste signaling through depletion of cells or signaling components, for instance, Gnat3^−/−^ mice lacking α‐gustducin, Pou2f3^−/−^ mice lacking SCCs, and Trpm5^−/−^ mice lacking TRPM5 [127]. Employing these lines can be used to confirm bitter taste receptor activation but cannot be used to elucidate the function of individual Tas2rs. Another option is the use of transgenic mice that express human TAS2Rs, as has previously been created for TAS2R38 and TAS2R16 [158]. These lines could account for variation in selectivity and sensitivity between humans and mice [158]. Furthermore, they would likely prove especially useful for human TAS2Rs lacking orthologous pairs in mice [158, 159].

## 3 Conclusion

Although originally recognized for their gustatory expression and harm‐avoidance properties, the extraoral functions of TAS2Rs have recently come into light. TAS2Rs are expressed in a variety of tissues, such as the upper respiratory tract, gastrointestinal tract, reproductive tissue, and the central nervous system (Table 1). Within these tissues, TAS2Rs are believed to function as sentinels in pathogen detection through recognition of bacterial‐derived QSMs. It is estimated that 1.91 million deaths will result from antimicrobial resistance by 2050, with a further 8.22 million associated deaths, demonstrating an urgent need for alternative interventions [160]. In recent years, there has been a shift in focus towards host‐pathogen interactions with the eventual goal being the creation of immunomodulatory‐based drugs. Research into the basic mechanisms through which TAS2Rs detect and respond to bacteria is a promising starting point. Future studies should place greater emphasis on in vivo models to confirm whether findings translate to living systems.

## Author Contributions

**Erin Rudolph:** conceptualization, writing – original draft, writing – review and editing. **Hannah Dychtenberg:** conceptualization, writing – original draft, writing ‐ review and editing. **Austin Pozniak:** conceptualization, writing – original draft, writing – review and editing. **Priyanka Pundir:** conceptualization, writing – review and editing, project administration, supervision, funding acquisition.

## Ethics Statement

The article is based on previously conducted studies and does not contain any new studies involving human participants or animals performed by any of the authors.

## Conflicts of Interest

The authors declare no conflicts of interest.

## Data Availability

Data sharing is not applicable to this article as no new data were created or analyzed in this study. All data supporting the findings of this study are available within the paper.
